# Development, validation, and dissemination of a simulated case-based diabetes learning tool for primary care residents and providers

**DOI:** 10.1186/1748-5908-10-S1-A37

**Published:** 2015-08-20

**Authors:** Heidi L Ekstrom, William A Rush, Patrick J O'Connor, JoAnn M Sperl-Hillen

**Affiliations:** 1 HealthPartners Institute for Education and Research, Minneapolis, MN 55425, USA

## Problem

Provider adherence to current diabetes care clinical guidelines is relatively low. We developed a virtual case-based Simulated Diabetes Education (SimDE) intervention to teach primary care residents how to implement current diabetes care clinical guidelines, and evaluated the impact of this tool in a randomized trial.

## Methods

Nineteen primary care residency programs, with 341 volunteer residents in all post-graduate years (PGY), were randomly assigned to a SimDE intervention group or control group (CG). The web-based interactive educational intervention utilized computerized virtual patients who responded to provider actions through programmed simulation models. Eighteen distinct learning cases (L-cases) were assigned to SimDE residents over 6 months. Impact was assessed using performance on four virtual assessment cases (A-cases), an objective knowledge test, and pre-post changes in diabetes knowledge and self-assessed confidence. Generalized linear mixed models were used to control for clustering of residents and baseline differences in knowledge.

## Results

The percentage of residents appropriately achieving composite clinical goals for glucose, blood pressure, and lipids on Assessment Cases was: Case 1, SimDE = 21.2%, CG = 1.8%, p=0.002; Case 2, SimDE = 15.7%, CG = 4.7%, p=0.02; Case 3, SimDE = 48.0%, CG = 10.4%, p < 0.001; Case 4, SimDE = 42.1%, CG = 18.7%, p=0.004. Change in knowledge score and self-assessed confidence were also significantly better for the SimDE than CG group.

## Implications for dissemination and implementation

Virtual case-based SimDE enhanced measures of resident performance, improved objective and subjective knowledge scores, and raised self-confidence in managing patients with diabetes. These results are congruent with studies of similar diabetes learning interventions in practicing physicians that demonstrated improved quality of diabetes care in real patients. Broader dissemination of this very scalable web-based simulated diabetes learning tool may improve the diabetes care capabilities of the present and future healthcare workforce.

